# Vibration Analysis of Aviation Electric Propulsion Test Stand with Active Main Rotor

**DOI:** 10.3390/s25216547

**Published:** 2025-10-24

**Authors:** Rafał Kliza, Mirosław Wendeker, Paweł Drozd, Ksenia Siadkowska

**Affiliations:** 1 Department of Technology Fundamentals, Faculty of Production Engineering, University of Life Sciences in Lublin, 13 Akademicka Street, 20-950 Lublin, Poland; rafal.kliza@up.lublin.pl (R.K.); pawel.drozd@up.lublin.pl (P.D.); 2 Department of Thermodynamics, Fluid Mechanics and Aviation Propulsion Systems, Faculty of Mechanical Engineering, Lublin University of Technology, 36 Nadbystrzycka Street, 20-618 Lublin, Poland; m.wendeker@pollub.pl; 3 Efficient Powertrain Solutions, School of Technology and Innovations, University of Vaasa, Wolffintie 32, 65200 Vaasa, Finland

**Keywords:** electric propulsion system, electric motor, piezoelectric accelerometer, vibration analysis, FFT, power spectral density, rotor blade

## Abstract

This paper focuses on the vibration analysis of a prototype helicopter rotor test stand, with particular attention to the dynamic response of its electric propulsion system. The stand is driven by an induction motor and equipped with composite rotor blades of various geometries, including blades with shape memory alloy (SMA)-based torsion actuators for angle of attack (AoA) adjustment. These variable geometries significantly influence the system’s dynamic behavior, where resonance phenomena may pose risks to structural integrity. The objective was to investigate how selected operational parameters specifically motor speed and AoA affect the vibration response of the propulsion system. Structural vibrations were measured using a tri-axial piezoelectric accelerometer system integrated with calibrated signal conditioning and high-resolution data acquisition modules. This setup enabled precise, time-synchronized recording of dynamic responses along all three axes. Fast Fourier Transform (FFT) and Power Spectral Density (PSD) methods were applied to identify dominant frequency components, including those associated with rotor harmonics and SMA activation. The highest vibration amplitudes were observed at an AoA of 16°, but all results remained within the vibration limits defined by MIL-STD-810H for rotorcraft drive systems. The study confirms the importance of sensor-based diagnostics in evaluating electromechanical propulsion systems operating under dynamic loading conditions.

## 1. Introduction

The implementation of electric propulsion systems (EPS) in rotary-wing aircraft is a promising direction for improving efficiency, safety, and environmental performance [1]. In recent years, research into hybrid and fully electric propulsion systems for helicopters and UAVs has accelerated due to advancements in electric motor efficiency, thermal management, and high-density energy storage [2].

Rotor systems are subject to complex aerodynamic and structural interactions. Dynamic coupling between cyclic blade loading, gearbox resonance modes, and control disk deflection can generate severe vibrations transmitted to the airframe. In extreme cases, this can lead to destructive resonance, threatening structural integrity. One emerging solution is the use of smart materials such as Shape Memory Alloys (SMA), which enable real-time control of blade twist or angle of attack (AoA) [3]. While SMA-based actuators improve aerodynamic adaptability, they also introduce new challenges related to vibration behavior, especially under varying operational loads.

Monitoring and analysis of such vibrations require high-fidelity sensing systems capable of capturing dynamic responses over a wide frequency range. Piezoelectric accelerometers, especially tri-axial ICP types, have proven effective in identifying rotor-induced harmonics, gearbox interactions, and high-frequency transients in rotorcraft. These sensors, when integrated with calibrated signal conditioning and data acquisition modules, form the foundation of modern vibration diagnostics. However, few studies address sensor-based vibration analysis in systems with actively controlled blade geometry using SMA actuators.

Test stand development typically begins with CAD modeling and structural verification using FEM [4,5]. This is typically supported by CFD simulations and numerical stability assessments [6,7,8]. Due to their efficiency and controllability, electric motors are favored in test rigs [9,10].

Research on propulsion systems often targets vibration phenomena in rotating blades within their operational speed ranges. High-speed cameras are used for visualizing vibration modes [11], while dynamic interactions in composite blades, especially under changing frequencies and amplitudes, lead to complex oscillatory patterns [12,13]. Effective damping strategies, including the Nonlinear Energy Sink (NES) approach, have demonstrated significant vibration reduction [14]. Additional methods such as the Single Equivalent Mass (SEM) and Transfer Matrix Method (TMM) have also been applied within the 152–500 Hz range [15].

Frequency-domain techniques like Fast Fourier Transform (FFT) and Power Spectral Density (PSD) remain standard tools for vibration analysis [16,17]. Experimental measurements are often combined with numerical simulations to validate dynamic models [18,19]. Mechanical vibrations are a key parameter in test stands using helicopter gearboxes. Bearing race damage may be masked by gear-related vibrations. To detect such faults, energy-based indicators [20] and the SANC method are selected for signal extraction. In studies of the SA330 MGB gearbox, planetary bearing faults were detected under varying load conditions [21]. High analytical accuracy is achieved using CSCD methods based on Toeplitz and cyclic matrices [22].

Complementary techniques such as acoustic emission (AE) allow detection of high-energy events in complex structures [23], while recent studies have also explored the use of hybrid real-virtual diagnostics and time-frequency analysis (e.g., CWT, AR models) for identifying damage signatures [24,25,26]. Selected examples even involve combustion engines [27,28], though the focus in this paper is on electric drive systems.

Recent studies confirm their robustness in structural health monitoring of aerospace systems, highlighting their accuracy, environmental resilience, and ease of integration in composite structures [29]. Furthermore, a comparative analysis of vibration sensor technologies emphasizes the critical role of piezoelectric sensors in condition monitoring applications, especially for rotating machinery [30].

In the context of rotor blade design, understanding the structural and vibrational behavior of composite aerospace components is critical. Prior studies have analyzed the dynamic response of composite spars [31] and provided detailed analysis of main rotor with controlled geometric twist [32]. The last reference publication introduced and described in detail the project that provided the data used in the analysis. The topics discussed can be approached from various perspectives, and vibration analysis of the test stand is one of the aspects that should be considered in this research due to its potentially significant impact.

The current literature lacks data on the effect of varying blade angle of attack (AoA) controlled by SMA actuators on the vibration response of rotor drive systems. The tested blades employ active actuators to geometrically twist the profile and vary AoA along the leading edge. There is a research gap concerning the interaction between AoA, rotor speed, and vibration behavior.

This paper aims to assess the impact of blade geometry variations on the dynamic behavior of a test stand with composite blades. The vibration signals are analyzed to evaluate how AoA changes affect system dynamics and the safety of active SMA-based blades. This study fills the research gap by investigating vibration responses of a test stand with composite rotor blades and electric propulsion under different RPM and AoA conditions. Using Fast Fourier Transform (FFT) and Power Spectral Density (PSD), the results are compared against MIL-STD-810H standards [33].

## 2. Research Object

### 2.1. Test Stand

The core of the test stand is an upper support frame, composed of a fixed cage and a movable top frame. The drive system includes a main gearbox with a swashplate and an AC electric motor. The blade angle of attack (AoA) was adjusted via an electromechanical linear actuator connected to the swashplate (range: 0° to 20°). The stand is equipped with a control and measurement system that enables remote manipulation of the geometric twist of the blades.

AoA adjustment is achieved by activating shape memory alloy (SMA) actuators integrated into each blade. These actuators are controlled by electric current and temperature. Operating parameters of the stand were recorded using an electronic measurement system.

**Figure 1:**
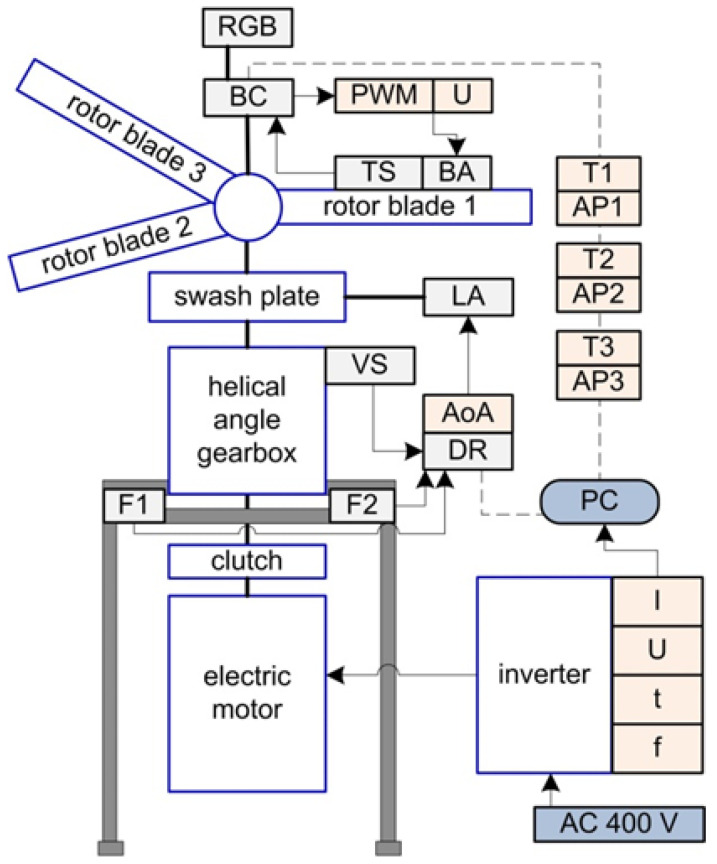
General scheme of the test stand, BC—blade controller, RGB—color signaling of status, TS—temperature sensor, BA—blade actuator, LA—linear actuator, VS—three axis vibration sensor, DR—data recorder, F1, F2—tensometer, T1–T3—temperature in blade, AP1–AP3—actuator position, AoA—angle of attack, PWM—actuator power signal filling, U—voltage, I—current, t—time, f—frequency. The arrows indicate the directions of the signal and the electric supply.

The test stand presented on Figure 1 is powered by a three-phase electric motor, with its voltage and current regulated by an inverter connected to the power grid. The operator can smoothly adjust the motor shaft speed at every test stage and can perform an emergency stop using a safety button. The electric motor drives the helicopter’s main gearbox via a clutch, which is connected to a rotor equipped with a swashplate. Prototype collective blades were mounted on the rotor. During testing, a range of parameters was recorded, including platform vibrations along the X, Y, and Z axes, lift force generated by the blades, and operating parameters of the SMA actuators. All data were continuously logged on a PC.

The test stand is powered by an electric motor operating at 1500 rpm. Vibration isolation of the test stand relative to the ground was ensured by placing the structure on four BSL 08012 isolators (MULTI Wyroby Gumowe, Jugowice, Poland). The shaft velocity is variably adjusted via the inverter control panel, and measurement data are transmitted to the PC using an RS485-USB converter. Detailed technical parameters are listed in Table 1.

### 2.2. Measuring System

**Figure 2:**
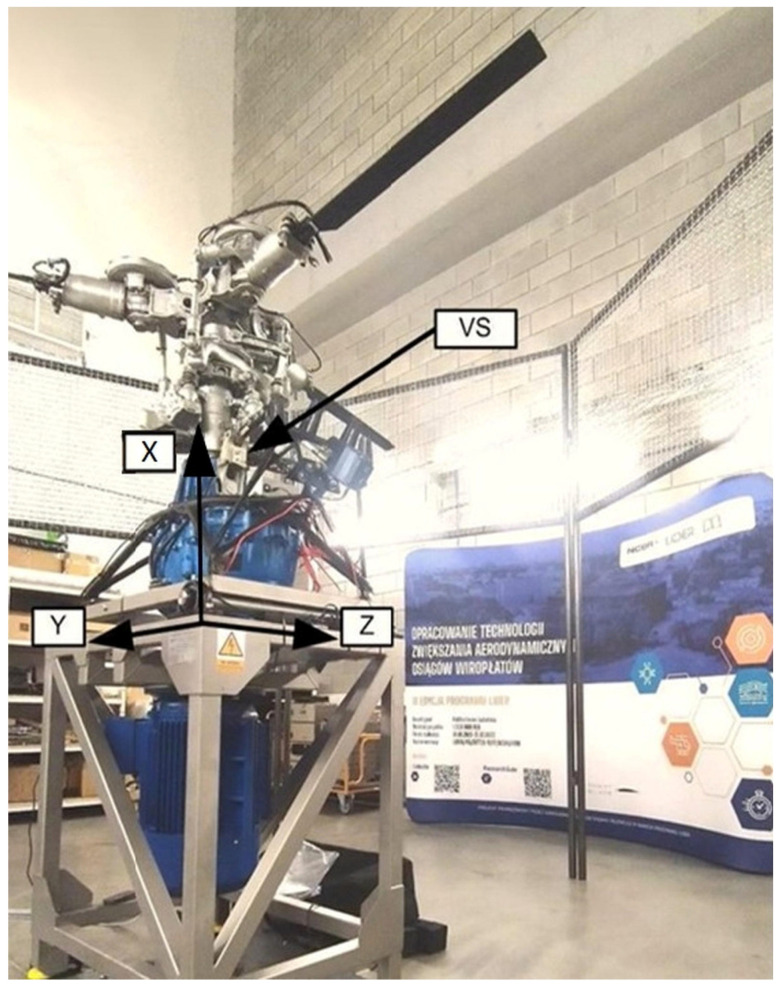
Axis and vibration sensors (VS) on the test stand.

**Figure 3:**
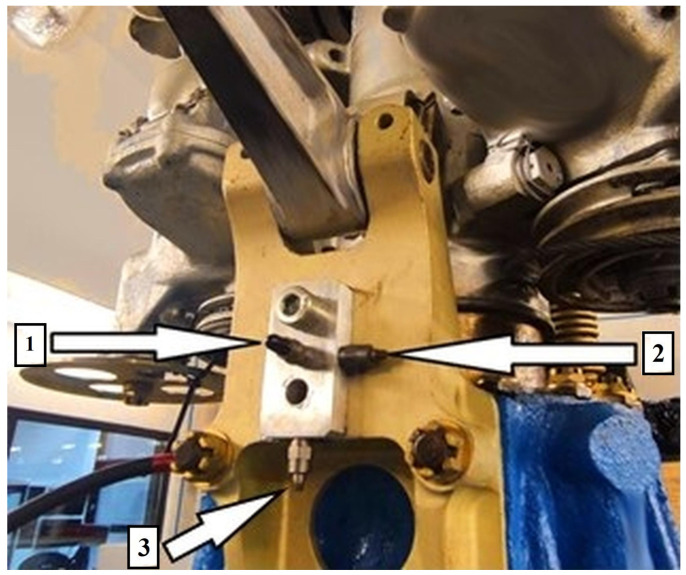
Placement of vibration sensors on the main gearbox: 1: Y-axis, 2: Z-axis, 3: X-axis.

Figure 2 indicates that the test stand enables vibration measurements in the X, Y, and Z axes. These axes were selected due to the lift force acting along the X-axis, while most rotating components move in the Y–Z plane. The stand is equipped with a specialized system for recording vibration waveforms. Vibration sensors were mounted at the top of the main gearbox housing and shown on Figure 3.

**Figure 4:**
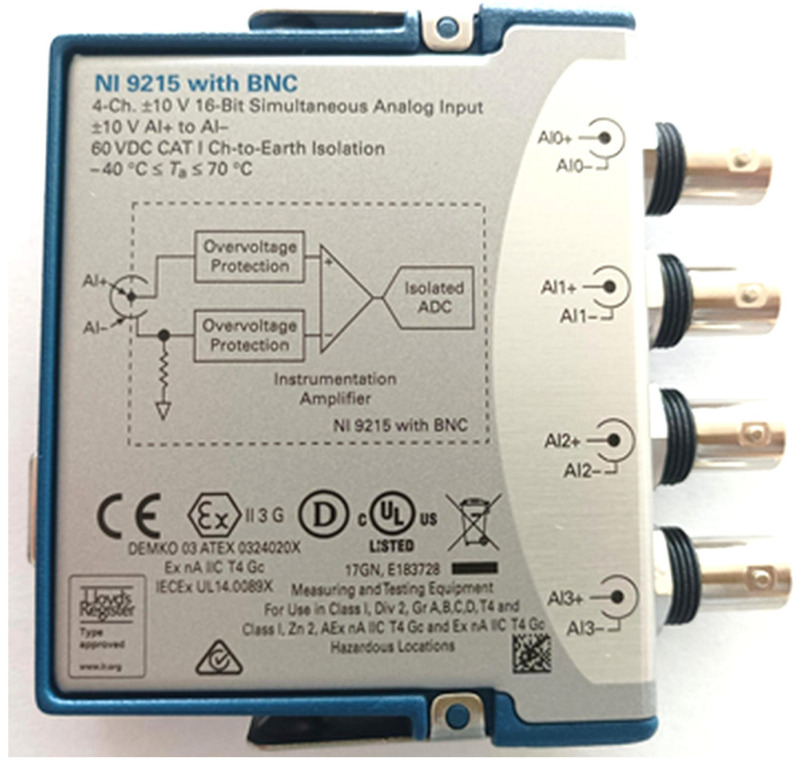
NI-9215 measuring card.

The vibration measuring system consists of the following devices:

1. A set of three piezoelectric vibration sensors M353B12 (PCB Piezotronics, Depew, NY, USA), showed in Figure 3, was used to record vibrations along all axes. The transverse sensitivity and nonlinearity of these sensors ensure accurate acquisition of dynamic responses across the frequency bands relevant to rotorcraft test stands.
2. Signal conditioning was performed using the VibAMPPA-3000 (EC Electronics Ltd., Basingstoke, United Kingdom) charge amplifier, which provided charge-to-voltage conversion, impedance matching, and preliminary low-pass filtering appropriate for ICP piezoelectric sensor signals.
3. Signal acquisition was performed using the NI-9215 C-Series (National Instruments, Austin, TX, USA) analog input module (Figure 4), equipped with four simultaneously sampled differential input channels.

The full sensor chain comprising the piezoelectric elements, analog conditioning, and digital acquisition was calibrated in accordance with the manufacturer’s specifications. The low-noise electronics and high sensitivity ensured robust signal quality, even in the presence of structural damping or aerodynamic noise. The vibration signal, represented as a voltage waveform, is processed by the NI-9215 module and streamed to the acquisition PC.

The description of the powertrain and rotor components is provided to give context for the vibration measurements presented in the next sections. The focus of this study is on the global dynamic response of the complete rotor blade test stand.

## 3. Test Results

### 3.1. FFT Analysis

#### 3.1.1. Measurement Setup and Preprocessing

The following results are presented to evaluate the vibration response of the complete test stand with SMA-equipped blades and to verify whether the measured levels comply with the vibration limits specified in MIL-STD-810H. The applied sensor system was crucial in capturing subtle changes in vibration behavior resulting from variations in blade AoA and rotor speed. In particular, the high directional accuracy of the tri-axial accelerometers allowed identification of dominant vibration modes along specific structural axes. The sensors also enabled differentiation between drive induced harmonics and localized modal interactions, facilitating interpretation of dynamic coupling effects within the rotor–gearbox–support assembly. Vibrations of the test stand were recorded along the X, Y, and Z axes. For each measurement point, data acquisition lasted 25 s. During the experiment, two independent variables were modified: the rotational speed of the electric motor’s drive shaft and the angle of attack of the main rotor blades. The blades were made of composite material with an asymmetric aerodynamic profile. Spectral data derived from the piezoelectric sensor output.

Three shaft rotational frequencies were selected for the study: f_0_ = 25 Hz, 50 Hz, and 70 Hz. For each frequency, the blade AoA was set to 0°, 8°, and 16°. The vibration sensors measured acceleration along three axes at a sampling rate of 1670 Hz. The recorded data were saved as acceleration vs. time functions.

**Figure 5:**
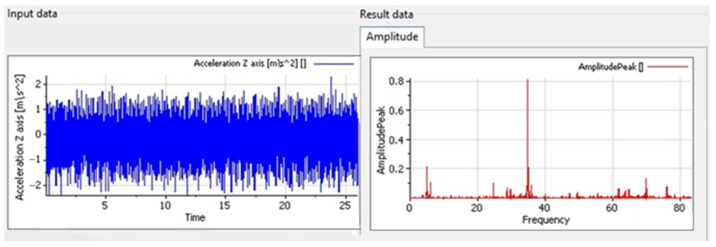
NI DIAdem 2023 Q2 software operational window.

The data underwent preliminary preprocessing before being imported into the NI DIAdem environment (Figure 5). Spectral analysis was performed using Fast Fourier Transform (FFT), which made it possible to obtain vibration spectra characteristic of the tested stand [34].

#### 3.1.2. Results at Lowest Rotor Velocity

**Figure 6:**
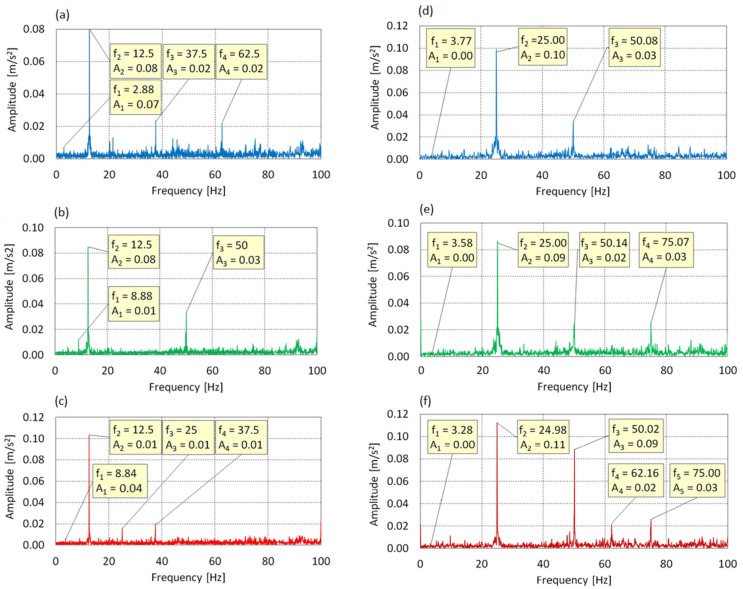
Vibration spectrum for the f_0_ = 25 Hz: (**a**) AoA = 0°, X-axis; (**b**) AoA = 8°, X-axis; (**c**) AoA = 16°, X-axis; (**d**) AoA = 0°, Y-axis; (**e**) AoA = 8°, Y-axis; (**f**) AoA = 16°, Y-axis.

**Figure 7:**
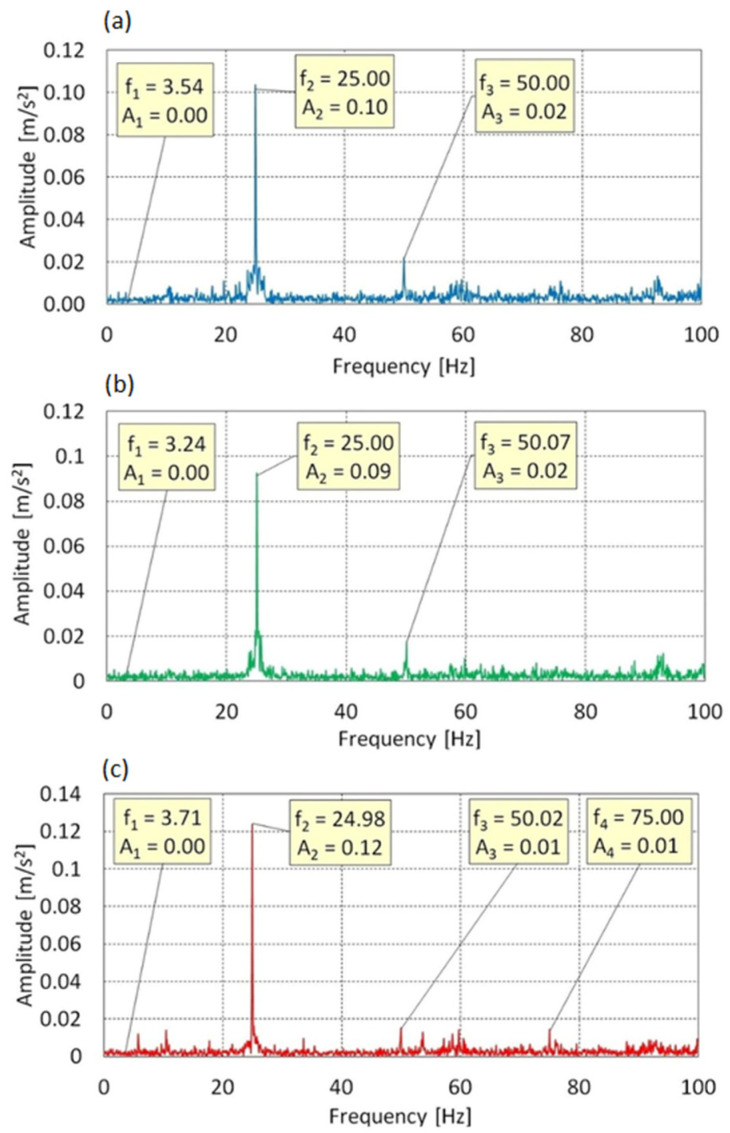
Vibration spectrum for the f_0_ = 25 Hz in Z-axis: (**a**) AoA = 0°, (**b**) AoA = 8°, (**c**) AoA = 16°.

Figure 6 and Figure 7 present the vibration spectra along the X, Y, and Z axes at a motor shaft speed of f_0_ = 25 Hz. The variable parameter was the blade angle of attack. The charts illustrate prominent peaks corresponding to the fundamental and harmonic frequencies of the drive shaft (f_0_ = 25 Hz) and the swashplate. A linear scale was chosen for the all FFT spectra to clearly display all significant frequency components without masking lower-amplitude peaks.

In the spectrum shown in Figure 6, four dominant components were identified: f_1_ = 2.88 Hz, f_2_ = 12.5 Hz, f_3_ = 37.5 Hz, and f_4_ = 62.5 Hz. The frequency f_1_ corresponds to the actual rotational speed of the main rotor, resulting from gear reduction (f_0_ = 25 Hz) with a ratio of 8.68:1. The highest amplitude (0.08 m/s^2^) occurred at f_2_ = 12.5 Hz, which is a subharmonic (0.5× f_0_). The presence of components f_3_ and f_4_, corresponding to 1.5× and 2.5× f_0_, indicates that the vibration spectra exhibit a rich frequency content with multiple harmonic and sub-harmonic components, reflecting the multi-source excitation of the test stand. The absence of a peak at f_0_ = 25 Hz suggests selective damping of the fundamental excitation and dominance of subharmonic resonance.

At AoA = 8°, the spectral structure changes—three main components appear: f_1_ = 8.88 Hz, f_2_ = 12.5 Hz, and f_3_ = 50 Hz. The frequency f_1_ may result from cyclic excitation of the three-bladed rotor. The presence of harmonics and a clear peak at 50 Hz indicates intensified dynamic activity due to increased aerodynamic loading.

At AoA = 16°, the spectrum becomes further dispersed four peaks are visible: f_1_ = 8.84 Hz, f_2_ = 12.48 Hz, f_3_ = 25 Hz, and f_4_ = 37.5 Hz. Amplitudes are relatively even, suggesting a transition from a subharmonic to a more harmonic system response, with a uniform distribution of energy among primary and secondary components.

Changes in the spectral structure with increasing AoA can be interpreted as an effect of growing aerodynamic loads. Excitation at approximately 3× f_rotor_ may be attributed to asymmetrical blade loading. Such frequency distributions are typical for systems with dynamic coupling between aerodynamic forces and structural response.

In the Y-axis spectrum, three dominant components were observed: f_1_ = 3.77 Hz, f_2_ = 25 Hz, and f_3_ = 50 Hz. The component f_1_ corresponds to rotor rotation but has minimal amplitude and does not significantly affect the dynamic response. The main energy is concentrated at f_2_ = 25 Hz (drive frequency), indicating direct energy transfer from the propulsion system to the structure. The component f_3_ = 2 × f_0_ is likely due to asymmetric excitation or nonlinear structural deformations.

The highest amplitude (0.10 m/s^2^) was recorded at f_3_ = 25 Hz. The absence of subharmonics and minimal contribution from higher harmonics suggests a harmonic and linear system response at AoA = 0°.

At AoA = 8°, the spectral structure broadens f_4_ = 75.07 Hz (3× f_0_) appears, along with a slight increase at f_3_ = 50.14 Hz. The presence of a third harmonic reflects growing system nonlinearity due to increased aerodynamic forces. Nevertheless, f_2_ = 25 Hz remains dominant, indicating a quasi-harmonic response.

At AoA = 16°, the spectrum becomes more complex besides f_2_ and its harmonics, a new component f_4_ = 62.16 Hz emerges, not being a multiple of f_0_. This may indicate local resonances or internal modal coupling. The increased amplitude of f_3_ relative to f_2_ suggests intensifying nonlinear behavior under full aerodynamic loading.

**Figure 8:**
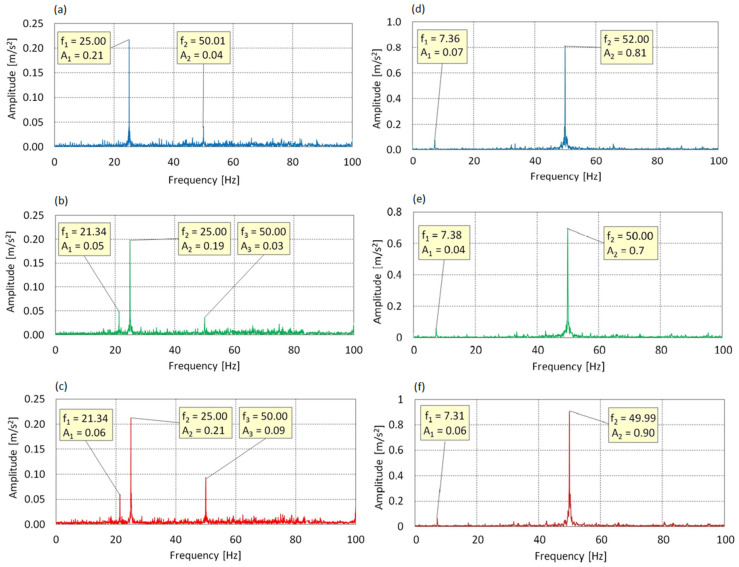
Vibration spectrum for the f_0_ = 50 Hz: (**a**) AoA = 0°, X-axis; (**b**) AoA = 8°, X-axis; (**c**) AoA = 16°, X-axis; (**d**) AoA = 0°, Y-axis; (**e**) AoA = 8°, Y-axis; (**f**) AoA = 16°, Y-axis.

**Figure 9:**
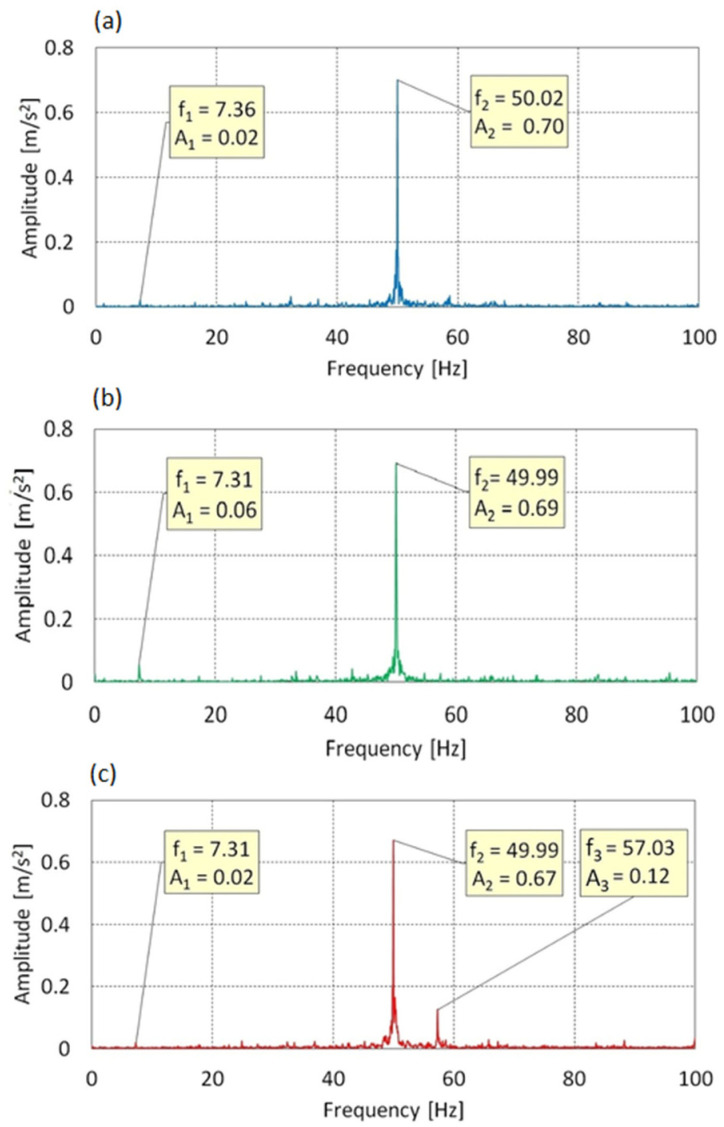
Vibration spectrum for the f_0_ = 50 Hz in Z-axis: (**a**) AoA = 0°, (**b**) AoA = 8°, (**c**) AoA = 16°.

**Figure 10:**
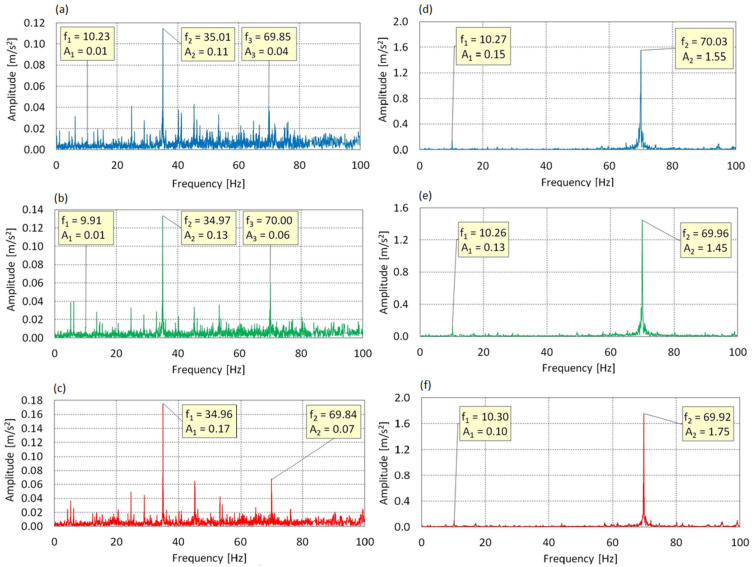
Vibration spectrum for the f_0_ = 70 Hz: (**a**) AoA = 0°, X-axis; (**b**) AoA = 8°, X-axis; (**c**) AoA = 16°, X-axis; (**d**) AoA = 0°, Y-axis; (**e**) AoA = 8°, Y-axis; (**f**) AoA = 16°, Y-axis.

**Figure 11:**
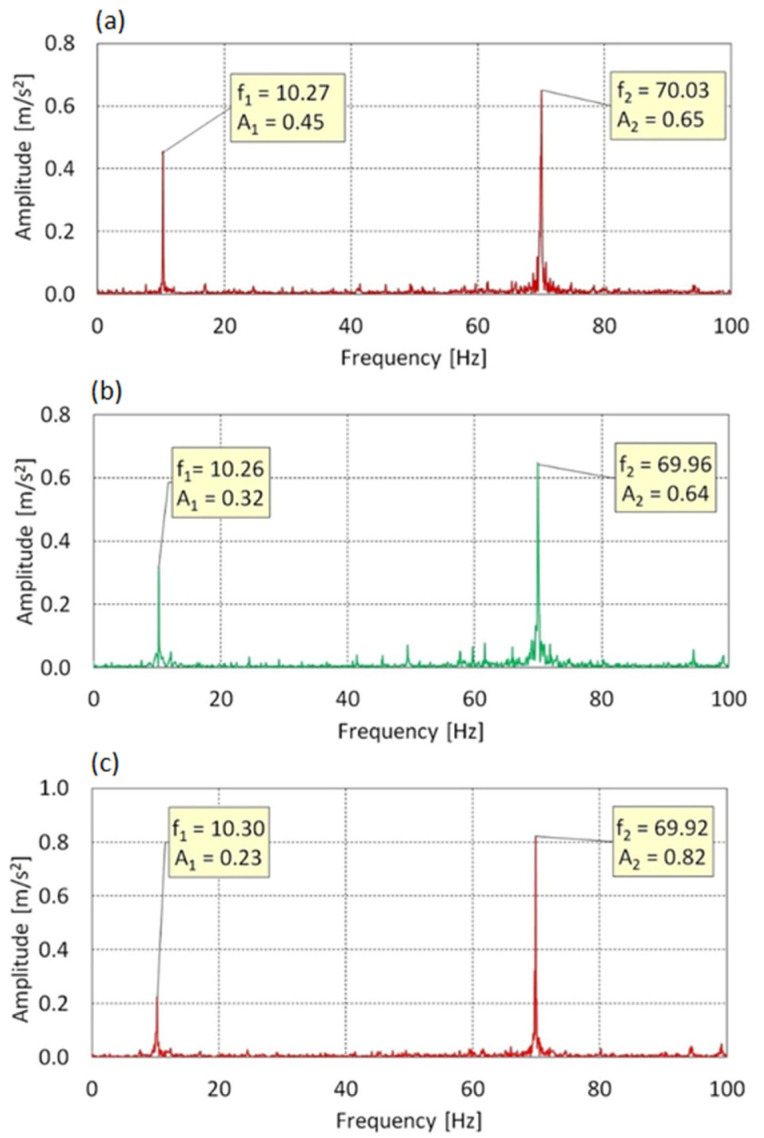
Vibration spectrum for the f_0_ = 70 Hz in Z-axis: (**a**) AoA = 0°, (**b**) AoA = 8°, (**c**) AoA = 16°.

Since vibrations along the Y-axis occur in the rotor plane, the observed increase in harmonic content and additional components with growing AoA is associated with increased lateral forces and complex structural responses due to unsteady aerodynamic conditions. The spectral peaks presented on Figure 6, Figure 7, Figure 8, Figure 9, Figure 10 and Figure 11 are classified based on their order dependence on shaft speed and gearbox ratio, allowing us to distinguish between drive-related and rotor-related harmonics.

Figure 7 presents the vibration spectra along the Z-axis. Three dominant components were identified: f_1_ = 3.54 Hz, f_2_ = 25.00 Hz, and f_3_ = 50.00 Hz. Component f_1_, associated with rotor rotation, showed minimal amplitude and did not significantly influence the dynamic response. The dominant component was f_2_ = 25 Hz (f_0_) with a maximum amplitude of 0.10 m/s^2^, while f_3_ = 2 × f_0_ had a much lower amplitude (0.02 m/s^2^), suggesting weak nonlinearities or asymmetric loads.

The lack of subharmonics and suppression of higher harmonics confirm the harmonic nature of vibrations at AoA = 0°. At AoA = 8°, the spectral structure remains largely unchanged f_0_ still dominates, and the influence of higher harmonics is negligible. Small frequency shifts may result from signal nonstationarity or sampling limitations.

At AoA = 16°, an additional component f_4_ = 75.00 Hz (3× f_0_) appears, but its amplitude remains low. Dominance of f_2_ = 24.98 Hz is preserved, confirming a stable dynamic response. The increase in higher harmonic amplitudes may signal early signs of nonlinearity, but does not conclusively indicate structural resonances.

Vibrations in the Z-axis lying in the rotor plane, are primarily induced by mechanical drive excitation. The spectral characteristics remain stable and linear, even under full aerodynamic loading.

#### 3.1.3. Results at Medium Rotor Velocity

Figure 8 and Figure 9 presents the vibration spectra of the test stand in the X, Y, and Z axes are presented for a rotational speed of the electric motor shaft equal to f_0_ = 50 Hz.

Figure 8 shows the frequency spectrum for an Angle of Attack of 0°. Two dominant components were identified: f_1_ = 25.00 Hz (A_1_ = 0.21 m/s^2^) and f_2_ = 50.01 Hz (A_2_ = 0.04 m/s^2^). Frequency f_1_, corresponding to 0.5× f_0_, exhibits energetic dominance, indicating the presence of subharmonic dynamic excitation. The relatively low amplitude of f_2_ (1× f_0_) may suggest a nonlinear system response, potentially resulting from gearbox backlash or excitation asymmetry.

At AoA = 8°, the spectrum expands to include an additional component f_1_ = 21.34 Hz (A_1_ = 0.05 m/s^2^), which may indicate modal resonance. Frequency f_2_ = 25 Hz remains dominant, while the contribution of the fundamental harmonic (f_3_ = 50 Hz) remains limited. The system maintains a subharmonic response profile, accompanied by increased structural activity.

At AoA = 16°, an increase in the amplitude of f_3_ = 50.02 Hz (A_3_ = 0.09 m/s^2^) is observed, while the dominance of f_2_ = 25 Hz is preserved. The persistence of component f_1_ = 21.34 Hz indicates continued dynamic coupling, and the elevated activity at 1× f_0_ is likely a result of full aerodynamic blade loading.

Vibrations in the X-axis, aligned with the direction of lift, are characterized by a dominant 0.5× f_0_ subharmonic and the presence of additional components, which may suggest nonlinear resonance phenomena. The increase in energy at f_0_ for higher AoA values is likely due to asymmetric thrust; however, the absence of amplitude escalation rules out the presence of flutter.

For the frequency spectrum along the Y-axis at AoA = 0°, two components were identified: f_1_ = 7.36 Hz (A_1_ = 0.07 m/s^2^) and f_2_ = 52.00 Hz (A_2_ = 0.81 m/s^2^). Frequency f_1_ exceeds twice the rotational speed of the main rotor, which may indicate cyclic excitation resulting from the geometry of the three-bladed configuration. The main source of dynamic excitation is f_2_ ≈ 1 × f_0_, confirming the dominant influence of the drive system.

At AoA = 8°, a similar spectral structure is maintained with f_1_ = 7.38 Hz and f_2_ = 50.00 Hz. The blade-related component remains stable, while the dominance of f_0_ continues with slightly reduced amplitude, possibly due to aerodynamic changes.

At AoA = 16°, the spectrum again consists of f_1_ = 7.31 Hz and f_2_ = 49.99 Hz, but a distinct increase in amplitude to A_2_ = 0.90 m/s^2^ is observed. This may result from intensified driving forces and moments transferred to the structure under full load conditions.

Vibrations along the Y-axis, which lies in the plane of rotor rotation, consistently exhibit dominance of f_0_ in all configurations. The presence of the 7.3–7.4 Hz band suggests cyclic aerodynamic excitation and potential modal activation. The dynamic response is harmonic in nature, with no signs of flutter-type instability, although the amplitude increase at AoA = 16° may indicate greater structural loading.

In Figure 9 for AoA = 0°, two spectral components were identified: f_1_ = 7.36 Hz, A_1_ = 0.02 m/s^2^ and f_2_ = 50.02 Hz, A_2_ = 0.70 m/s^2^. Component f_2_, matching the excitation frequency f_0_, dominates in terms of energy and clearly indicates strong transmission of drive-induced forcing in the plane of rotation. Frequency f_1_ remains consistent with the rhythm of the three-bladed rotor approximately 1.3× f_rotor_ but its energy contribution is negligible.

For AoA = 8°, the same spectral pattern is maintained: f_1_ = 7.31 Hz, A_1_ = 0.06 m/s^2^; f_2_ = 49.99 Hz, A_2_ = 0.69 m/s^2^. Both peaks remain at their positions, and the slight increase in amplitude of f_1_ may indicate a transient amplification of cyclic aerodynamic excitation. The spectrum remains stable and strongly harmonic.

At AoA = 16°, an additional component appears: f_3_ = 57.03 Hz, A_3_ = 0.12 m/s^2^, which may suggest the occurrence of modal resonance or a higher-order structural response mode. Component f_2_ continues to dominate, while the amplitude of A_1_ returns to its initial level.

In summary, vibrations along the Z-axis exhibit a clearly harmonic character and are strongly driven by the excitation frequency. The low activity of blade-related frequencies and the limited growth of additional amplitudes indicate a stable dynamic response with no signs of aeroelastic instability.

#### 3.1.4. Results at Highest Rotor Velocity

In the frequency spectrum shown in Figure 10 for the X-axis at AoA = 0°, five dominant components were identified: f_1_ = 12.53 Hz, f_2_ = 28.15 Hz, f_3_ = 38.19 Hz, f_4_ = 47.92 Hz, and f_5_ = 66.92 Hz. The spectrum does not exhibit a clear dominance of the fundamental drive frequency f_0_ = 70 Hz or its simple harmonics. The presence of strong peaks at irregular intervals suggests a complex, nonlinear dynamic response of the system along the axis of lift force.

At AoA = 8°, the spectral structure becomes even more dispersed. Six dominant components were recorded: f_1_ = 14.85 Hz, f_2_ = 27.26 Hz, f_3_ = 39.55 Hz, f_4_ = 49.90 Hz, f_5_ = 56.58 Hz, f_6_ = 64.76 Hz. The presence of energy bands within the 25–65 Hz range and the lack of a distinct peak at 70 Hz suggest unstable energy transfer and possible excitation of local vibration modes.

At AoA = 16°, a similarly complex spectral structure is observed. The most relevant frequencies are: f_1_ = 12.86 Hz, f_2_ = 24.82 Hz, f_3_ = 39.07 Hz, f_4_ = 48.36 Hz, f_5_ = 57.43 Hz, f_6_ = 66.41 Hz. The distribution of frequencies and amplitudes remains irregular, and the dominant peaks do not correlate with f_0_ or its harmonics. This may indicate dynamic decoupling of the drive from the structure or the onset of structural instability.

Considering that the analyzed vibrations occur along the X-axis, which corresponds to the direction of aerodynamic lift force, the multipoint distribution of spectral energy indicates irregular aerodynamic interactions and excitation of higher-order modes.

In the spectrum of the Y-axis at AoA = 0°, two clearly dominant components were identified: f_1_ = 10.27 Hz, A_1_ = 0.15 m/s^2^ and f_2_ = 70.03 Hz, A_2_ = 1.55 m/s^2^. Frequency f_2_ coincides with the fundamental drive frequency f_0_ = 70 Hz and exhibits the highest amplitude in the entire spectrum, clearly indicating that the primary dynamic excitation is transmitted by the drive shaft in the rotor’s plane of rotation. Frequency f_1_, close to four times the main rotor rotational speed f_rotor_ ≈ 8.06 Hz, may be associated with cyclic aerodynamic excitation of the three-bladed rotor or local support resonance.

At AoA = 8°, the spectral structure remains virtually unchanged. The same two components are present: f_1_ = 10.26 Hz, A_1_ = 0.13 m/s^2^ and f_2_ = 69.96 Hz, A_2_ = 1.45 m/s^2^. The slight reduction in the amplitude of the primary harmonic is likely due to changes in the aerodynamic force distribution or dynamic damping effects.

At AoA = 16°, both components are again observed, with a noticeable increase in the amplitude of the main peak: f_1_ = 10.30 Hz, A_1_ = 0.10 m/s^2^. The amplitude growth at f_0_ = 70 Hz may reflect intensified drive-induced vibrations caused by the increasing aerodynamic moment transmitted through the rotor structure under full loading.

Considering that vibrations along the Y-axis describe the dynamic response in the rotor plane, the presence and dominance of f_0_ = 70 Hz across the entire range of angles of attack confirm stiff and harmonic transmission of excitation from the drive. The stability of frequency f_1_ = 10.30 Hz and its low energy contribution suggest that potential cyclic effects are secondary to the main shaft excitation. The spectrum shows no signs of nonlinear instability or flutter in this direction.

In Figure 11 the vibration spectrum along the Z-axis lying in the plane of rotor rotation is characterized by the presence of two dominant components across all angles of attack: f_1_ = 10.27 Hz and f_2_ = 70.03 Hz. Component f_1_ corresponds directly to the rotational speed of the main rotor, indicating effective transmission of cyclic aerodynamic excitation to the structure in the Z-axis direction. Component f_2_, associated with the shaft rotation frequency f_0_ = 70 Hz, constitutes the primary source of vibrations in all cases.

For AoA = 0°, the amplitudes of the components are A_1_ = 0.45 m/s^2^ and A_2_ = 0.65 m/s^2^, respectively, suggesting a comparable contribution from both excitation sources. As the angle of attack increases to 8° and 16°, the contribution of f_1_ gradually decreases from A_1_ = 0.32 m/s^2^ to A_1_ = 0.23 m/s^2^, while the energy associated with the drive component f_2_ increases, reaching A_2_ = 0.82 m/s^2^ at AoA = 16°.

This trend suggests that under full aerodynamic loading, vibrations induced by the motor become dominant, while the influence of cyclic rotor rotation is partially suppressed. The system maintains a stable, harmonic dynamic response, with no signs of parasitic resonances or aeroelastic instability.

#### 3.1.5. Summary

Table 2, Table 3 and Table 4 present a summary of the vibration parameters of the gearbox housing in the test rig. The data show that for the Y and Z axes, vibration amplitudes recorded using calibrated accelerometers exhibit similar values at the same frequencies f_1_, f_2_, and f_3_. This suggests that the test stand has a proper mass distribution and is mounted with structural stability. Blank fields in the table indicate cases where no additional dominant frequency peaks were identified above the defined detection threshold. These omissions result from the absence of spectral components with amplitudes exceeding the noise floor or satisfying the established significance criteria within the analyzed frequency range. The varying number of detectable peaks under different test conditions reflects the dynamic nature of the system and the influence of rotor speed, blade geometry, and aerodynamic loading on the excitation of structural modes. As a result, only the most prominent peaks (typically above 0.01 m/s^2^) are reported, while weaker or less significant components are omitted for clarity and to maintain consistency with the FFT peak identification methodology. This approach ensures that the analysis remains focused on meaningful features of the vibrational spectrum, avoiding overinterpretation of low-amplitude noise artifacts.

Three measurement points were adopted during the tests, corresponding to the operating frequencies of the electric motor drive shaft: 25 Hz, 50 Hz, and 70 Hz. The selection of these values was based on the full operational range of the main gearbox. The frequency of 25 Hz represents the minimum rotational speed of the system, at which the lowest lift force is generated. The 50 Hz value serves as a reference, corresponding to the typical rotational speed of the main rotor during level flight of the helicopter from which the tested drive system originates. In contrast, 70 Hz represents the maximum allowable main rotor speed, consistent with the operational design assumptions of the structure.

The blade angle of attack (AoA) values 0°, 8°, and 16° were determined based on prior computational fluid dynamics (CFD) simulations conducted for the analyzed blade profile. Based on the recorded acceleration time histories and the performed Fourier transforms (FFT), frequency spectra were obtained to describe the dynamic response of the system. Spectral analysis allowed for the determination of the amplitude distribution as a function of frequency and the identification of potential resonance regions. The results obtained made it possible to evaluate the influence of measurement parameters such as drive shaft speed and blade angle of attack on the vibration characteristics of the test stand. Furthermore, the FFT analysis provided insights into the performance of selected system components, enabling an indirect estimation of the total transmission ratio between the active shaft and the main rotor swashplate.

### 3.2. PSD Analysis

#### 3.2.1. Investigation Procedures

To evaluate acceptable vibration levels, a Power Spectral Density (PSD) analysis was conducted. Reference was made to the American standard MIL-STD-810H [33], widely used in the defense, aerospace, and aviation industries for environmental qualification of military equipment. This standard includes detailed test procedures and standardized mechanical load profiles (including vibration), used in the design and structural validation of critical system components. Due to the lack of explicit civil standards addressing ground-based helicopter drivetrain test stands, the adoption of MIL-STD-810H as a reference is a justified and widely accepted engineering practice, enabling assessment of structural resilience according to rigorous operational requirements.

In this study, the data from Table 514.8D-IIIb were used, assigned to the category “Helicopter vibration exposure-On/Near drive system elements,” which covers the vibrational environment in close proximity to gearboxes, shafts, and structural supports of the propulsion system. This profile provides reference values of power spectral density (PSD) in units of g^2^/Hz as a function of frequency, with a specified peak limit band.

As part of the analysis, the reference limit points from the standard were implemented in the form of a digital step function. These were then linearly interpolated over the full 0–100 Hz frequency range, enabling direct comparison with the measured PSD spectrum obtained from recorded signals. The reconstructed vibration limit profile formed the basis for evaluating the compliance of the system’s dynamic response with operational requirements.

#### 3.2.2. Results at Lowest Rotor Velocity

**Figure 12:**
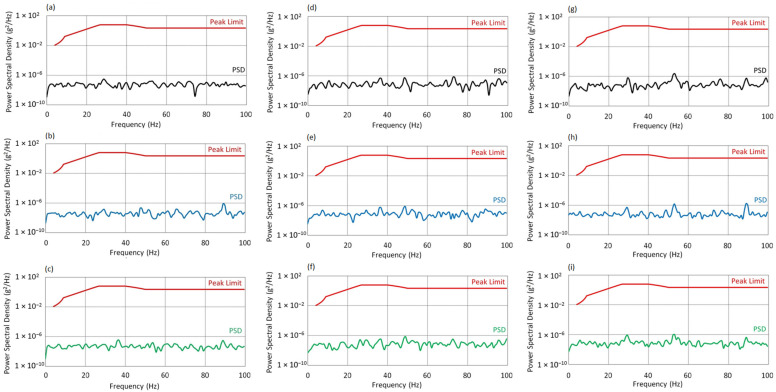
Vibration spectrum for the f_0_ = 25 Hz: (**a**) AoA = 0°, X-axis; (**b**) AoA = 8°, X-axis; (**c**) AoA = 16°, X-axis; (**d**) AoA = 0°, Y-axis; (**e**) AoA = 8°, Y-axis; (**f**) AoA = 16°, Y-axis; (**g**) AoA = 0°, Z-axis; (**h**) AoA = 8°, Z-axis; (**i**) AoA = 16°, Z-axis.

Figure 12 presents the Power Spectral Density spectra for the excitation frequency f_0_ = 25 Hz. For the X axis, the PSD spectra at f_0_ = 25 Hz for all angles of attack (AoA) remain below the threshold defined by the MIL-STD-810H standard. A logarithmic scale was applied to the PSD plots to enhance the visibility of low-amplitude components over a wide dynamic range.

- At AoA = 0°, moderate vibrations occur within the 10–60 Hz range, with local maxima around 30–40 Hz. However, their amplitudes are significantly lower than the permissible limits. The dynamic response is linear and stable.
- At AoA = 8°, a slight increase in energy is observed in the 20–60 Hz range, along with local enhancements above 80 Hz. Nevertheless, PSD values remain well below the standard threshold, and the system exhibits only moderate dynamic activity without signs of overload.
- At AoA = 16°, the spectral character remains largely unchanged. The vibration levels stabilize across a broad frequency range, and the system maintains a controlled, harmonic operating mode even under full aerodynamic load.

For the Y axis, the PSD spectra for all AoA values also remain significantly below the threshold values defined by the MIL-STD-810H standard.

- At AoA = 0°, the absence of distinct peaks indicates a stable structural response, which may result from effective vibration isolation in this configuration.
- At AoA = 8°, the spectrum shows more local maxima in the 20–60 Hz range, which may suggest the onset of resonant excitation or increased complexity of the dynamic response.
- At AoA = 16°, the spectral structure further develops; however, the amplitudes remain within safe limits. The increase in vibration energy indicates moderate intensification of dynamic interactions without exceeding safety margins.

For the Z axis, at f_0_ = 25 Hz, the PSD spectra remain low in all cases.

- At AoA = 0°, the lowest energy level was recorded absence of significant peaks confirms a well-damped, harmonic system response.
- At AoA = 8°, minor components appear around 55 Hz and 90 Hz, yet their amplitudes remain very low. This may signal the onset of structural activity.
- At AoA = 16°, the spectrum becomes more dispersed, especially above 50 Hz. Despite the increase in PSD levels, the system still meets the standard’s criteria, although the intensification of dynamic interactions may warrant further investigation.

#### 3.2.3. Results at Medium Rotor Velocity

**Figure 13:**
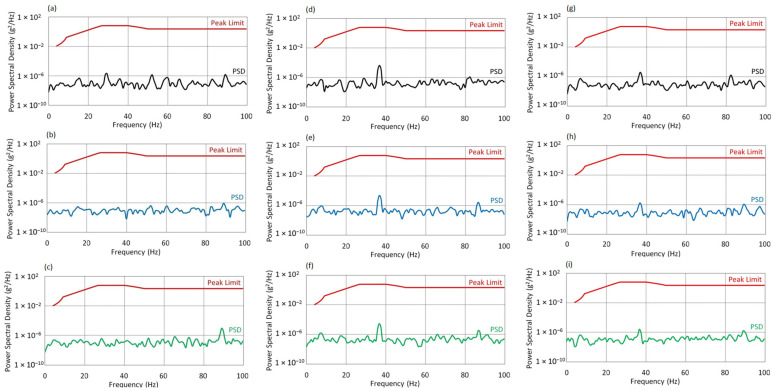
Vibration spectrum for the f_0_ = 50 Hz: (**a**) AoA = 0°, X-axis; (**b**) AoA = 8°, X-axis; (**c**) AoA = 16°, X-axis; (**d**) AoA = 0°, Y-axis; (**e**) AoA = 8°, Y-axis; (**f**) AoA = 16°, Y-axis; (**g**) AoA = 0°, Z-axis; (**h**) AoA = 8°, Z-axis; (**i**) AoA = 16°, Z-axis.

Figure 13 presents the Power Spectral Density spectra for the X, Y, and Z axes at an excitation frequency of f_0_ = 50 Hz, for three angles of attack. For the X axis at f_0_ = 50 Hz, the PSD spectra for all angles of attack remain below the permissible levels defined by the MIL-STD-810H standard.

- At AoA = 0°, the spectrum exhibits a compact structure with local maxima around 30, 55, and 90 Hz. The dynamic response is stable and well-controlled under nominal conditions.
- At AoA = 8°, the spectral range broadens into the 40–80 Hz band. The increase in amplitude around 90 Hz may result from the growing aerodynamic moment; however, PSD values remain safely within limits.
- At AoA = 16°, the spectrum becomes the most developed. The most noticeable amplitude increase is again observed near 90 Hz, which may indicate the activation of additional structural modes under full aerodynamic load. Despite this, vibration levels remain below critical thresholds.

For the Y axis at f_0_ = 50 Hz, the system exhibits characteristic dynamic behavior with increasing AoA.

- At AoA = 0°, a pronounced maximum appears in the 35–40 Hz band, with a dominant peak around ~37 Hz, reaching values exceeding 1 × 10^−6^ g^2^/Hz. Despite this localized intensification, the amplitude remains below the standard’s threshold.
- At AoA = 8°, the energy level in this range slightly decreases. This may be interpreted as the result of minor damping due to altered aerodynamic conditions or redistribution of forces within the structure.
- At AoA = 16°, no further changes in spectral distribution are observed. The consistent amplitude level suggests stable resonance conditions, regardless of increased angle of attack.

For the Z axis at f_0_ = 50 Hz, the main vibration energy is concentrated in the 30–40 Hz and 90–100 Hz bands, with a dominant peak in the 36–37 Hz region.

- At AoA = 0°, the amplitudes are highest but maintain a clear margin below the MIL-STD threshold.
- As the angle of attack increases (AoA = 8° and 16°), a systematic decrease in PSD levels is observed both in the resonance region and across the entire analyzed spectrum. This may indicate increased system stiffness or a change in vibration propagation conditions under higher aerodynamic loads.
- In all cases, the dynamic response in the Z-axis remains safe and shows no signs of exceeding acceptable vibration levels.

#### 3.2.4. Results at Highest Rotor Velocity

**Figure 14:**
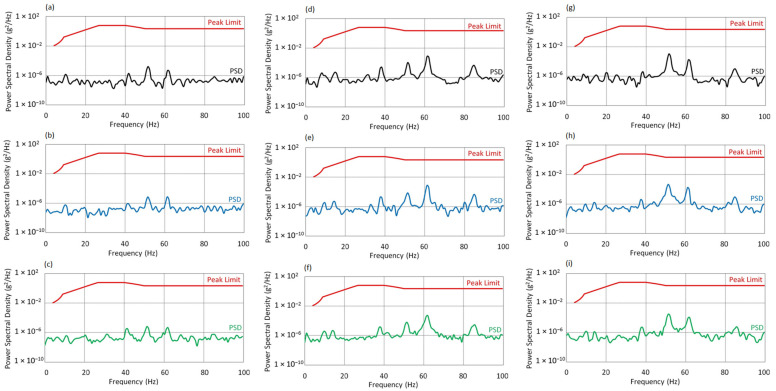
Vibration spectrum for the f_0_ = 70 Hz: (**a**) AoA = 0°, X-axis; (**b**) AoA = 8°, X-axis; (**c**) AoA = 16°, X-axis; (**d**) AoA = 0°, Y-axis; (**e**) AoA = 8°, Y-axis; (**f**) AoA = 16°, Y-axis; (**g**) AoA = 0°, Z-axis; (**h**) AoA = 8°, Z-axis; (**i**) AoA = 16°, Z-axis.

Figure 14 presents the Power Spectral Density spectra for the X, Y, and Z axes at an excitation frequency of f_0_ = 70 Hz, for three angles of attack. For the X axis at f_0_ = 70 Hz, in contrast to lower rotational speeds, dominant PSD amplifications occur in the 50–60 Hz range. The presence of two distinct peaks may indicate neighboring resonance frequencies, suggesting a complex modal structure of the system.

- At AoA = 0°, the PSD values remain well below the standard’s threshold.
- At AoA = 8°, a moderate decrease in amplitude is observed, whereas at AoA = 16°, the spectrum becomes more dispersed, and local maxima are further attenuated. This may indicate increased damping or a favorable shift in the system’s dynamic characteristics. Despite the presence of secondary resonances, the overall PSD remains within the MIL-STD-810H limits.

For the Y axis, at the same excitation frequency (f_0_ = 70 Hz), three main peaks are observed in the 38–62 Hz range.

- At AoA = 0°, the dominant component occurs at 62 Hz, which may result from structural resonance excitation or secondary effects from the drive system. The spectral profile suggests significant energy interaction near the f_0_ frequency. Although the harmonic structure is denser, amplitudes remain below threshold values.
- At AoA = 8°, the main components remain within the same frequency intervals, confirming the stability of the dynamic response.
- At AoA = 16°, a noticeable increase in PSD is observed in the 30–50 Hz band, indicating intensified energy transfer from the drive to the structure. The spectrum becomes more dispersed, yet remains within the limits defined by the standard.

For the Z axis, at AoA = 0°, two distinct peaks appear in the 50–55 Hz and 80–90 Hz regions. Although vibration energy levels rise compared to lower rotational speeds, the PSD values remain significantly below permissible limits. The spectral distribution suggests the presence of modal excitations.

- At AoA = 8°, the spectral characteristics are similar, though shifts and densification of components in the 55–65 Hz range are visible. This may result from a varying distribution of centrifugal and aerodynamic forces.
- At AoA = 16°, an amplitude increase is observed around 70 Hz, and the spectrum becomes more complex. The appearance of minor peaks above 80 Hz may indicate the activation of additional structural modes. Despite the increased spectral complexity, PSD levels remain safely within the MIL-STD-810H compliance range.

#### 3.2.5. Summary

The high resolution of the collected acceleration signals made it possible to capture both dominant harmonic components and subtle subharmonic or broadband effects. This demonstrates that the applied piezoelectric sensor configuration was sufficiently sensitive and dynamically capable of resolving the full range of system responses under varying load conditions. Fractional harmonics (0.5×, 1.5×, 2.5×) observed in the measured spectra are shown on the FFT plots (Figure 6, Figure 7, Figure 8, Figure 9, Figure 10 and Figure 11) to provide a complete representation of the vibration response of the test stand.

Causes of occurrence fractional components are related to nonlinear phenomena in the powertrain and blade excitation. Detailed identification of their origin is outside the scope of this work. Notably, the clear separation of frequency peaks corresponding to the rotor, gearbox, and swashplate motion indicates minimal sensor cross-talk and confirms the suitability of the instrumentation for structural vibration studies in rotorcraft systems. The repeatability and bounded amplitudes of the FFT spectra at all measurement points indicate that the system operates in a dynamically stable regime. PSD analysis confirmed that the vibration levels remain below the permissible limits defined by MIL-STD-810H, demonstrating that the test stand is not subjected to excessive vibration exposure.

## 4. Conclusions

The study was conducted on a rotor test bench designed to evaluate the global vibration response of the complete system, including the electric propulsion powertrain and prototype rotor blades. Three shaft rotation frequencies of the electric motor were selected (25 Hz, 50 Hz, and 70 Hz), along with three blade angles of attack (0°, 8°, 16°), adjusted using shape memory alloy (SMA) actuators. Vibration measurements were carried out using accelerometers placed along three axes: X (lift direction), Y, and Z (rotor plane). The acquired data were analyzed using the Fast Fourier Transform (FFT) and by calculating the Power Spectral Density (PSD).

- The following conclusions were drawn based on the FFT analysis: For f_0_ = 25 Hz: In the X axis, the dominant component was the subharmonic 12.5 Hz (0.5× f_0_), with no distinct peak at the base excitation frequency f_0_ = 25 Hz, indicating selective suppression of the primary forcing frequency. The dynamic response in the Y and Z axes was characterized by the stable presence of the drive frequency and its harmonics, with no signs of instability.
- For f_0_ = 50 Hz: The X axis continued to show dominance of the subharmonic (25 Hz), although an increase in amplitude was observed at f_0_ = 50 Hz for AoA = 16°. In the Y and Z axes, a stable spectral structure was identified with dominance of harmonics and blade-related signals around 7.3 Hz. Minor peaks, such as 57 Hz in the Z axis, were also detected, which may indicate local modal resonances.
- For f_0_ = 70 Hz: The spectrum in the X axis exhibited a dispersed nature with multiple dominant components, but without a clear presence of f_0_. This could suggest the development of dynamic instability. In the Y and Z axes, the f_0_ = 70 Hz component and a blade-related frequency around 10.3 Hz were dominant, with amplitudes increasing with AoA, indicating greater structural stiffness and direct energy transfer from the drive system to the structure.
- Key findings from the PSD analysis include: In all cases, the PSD spectra remained below the permissible thresholds defined by the MIL-STD-810H standard, confirming safe operating conditions.
- At the lowest speed (f_0_ = 25 Hz), energy distribution was uniform, particularly in the Z axis, where PSD levels were the lowest.
- With increasing drive speed, especially at 70 Hz, a higher density of peaks and increased PSD amplitudes were observed, primarily in the Y and Z axes, which may indicate the activation of additional dynamic modes.
- The X axis exhibited the highest spectral instability at 70 Hz, there was no clear dominance of f_0_, and the system response appeared dispersed and nonlinear.
- In the Y and Z axes, the drive component was dominant, and the PSD profiles were harmonic in nature, indicating effective energy transmission and structural rigidity in the rotor plane.

The sensor instrumentation exhibited consistent performance, maintaining high stability and repeatability throughout the entire range of test conditions. No signal saturation or clipping was observed, even at the highest rotor speeds and AoA configurations. This suggests that the measurement chain comprising high-grade piezoelectric elements and industrial-grade acquisition hardware provided reliable input for frequency-domain analysis. The robust setup offers potential for adaptation in active health monitoring or vibration-based diagnostic systems in aerospace applications.

The integration of PCB M353B12 accelerometers, VibAMPPA-3000 amplifier, and NI-9215 DAQ module created a high-fidelity measurement chain with wide dynamic range, excellent resolution, and minimal signal distortion. Each sensor channel was time-synchronized, gain-calibrated, and protected from aliasing and saturation. The system’s combined performance enabled confident identification of harmonic, subharmonic, and modal vibration components under varying AoA and motor speed conditions, with minimal risk of measurement artifacts or cross-talk.

In summary, the experiments were conducted within a safe frequency range. No indications of resonance or flutter were observed. Variation in motor frequency and blade angle of attack did not induce adverse dynamic effects. The highest vibration amplitudes recorded using calibrated accelerometers were recorded at f_0_ = 70 Hz, which may be attributed to mechanical imbalance within the motor–coupling–gearbox assembly. The observed fractional harmonics highlight the complex nature of the system’s vibration response. Increasing the angle of attack resulted in a reduction in vibration energy generated by the main rotor. Individual modes were identified using the analysis of the dependence of frequency on rotational speed. Components that are multiples of the shaft speed were assigned to drive forces. Comparison with the gear ratio model allows for the rotational frequency of the main rotor to be distinguished from the drive frequency.

Despite increased vibrations with rising rotational velocity, amplitude values remained below the limits defined in the MIL-STD-810H military standard. The test bench demonstrates high structural resilience, confirming its suitability for further research on rotor blades including those with greater pitch angles or lengths. Investigated prototype test stand, based on industrial helicopter components, enables safe and accurate measurements of forces and accelerations with high repeatability.

## Figures and Tables

**Table 1:** Technical parameters of the motor.

Parameter	Unit	Value
Model	-	YE3 220L-4
Power	kW	37
Asynchronous motor shaft speed	rpm	1480
Current at triangle connection 400 V	A	66.9
Efficiency class	-	IE3
Power factor	-	0.85
Rated torque	Nm	238.8
Weight	kg	328

**Table 2:** Summary table of vibration parameters of the test rig in the X-axis.

f_0_ (Hz)	AoA (°)	f_1_ (Hz)	A_1_ (m/s^2^)	f_2_ (Hz)	A_2_ (m/s^2^)	f_3_ (Hz)	A_3_ (m/s^2^)	f_4_ (Hz)	A_4_ (m/s^2^)
25	0	2.88	0.007	12.50	0.08	37.50	0.02	62.5	0.02
	8	8.88	0.01	12.50	0.08	50.00	0.03	-	-
	16	8.84	0.004	12.48	0.01	25.00	0.01	37.5	0.01
50	0	25.00	0.21	50.01	0.04	-	-	-	-
	8	21.34	0.05	25.00	0.19	50.00	0.03	-	-
	16	21.34	0.06	25.00	0.21	50.00	0.09	-	-
70	0	10.23	0.01	35.01	0.11	69.85	0.04	-	-
	8	9.91	0.01	34.97	0.13	70.00	0.06	-	-
	16	34.96	0.17	69.84	0.07	-	-	-	-

**Table 3:** Summary table of vibration parameters of the test rig in the Y-axis.

f_0_ (Hz)	AoA (°)	f_1_ (Hz)	A_1_ (m/s^2^)	f_2_ (Hz)	A_2_ (m/s^2^)	f_3_ (Hz)	A_3_ (m/s^2^)	f_4_ (Hz)	A_4_ (m/s^2^)	f_5_ (Hz)	A_5_ (m/s^2^)
25	0	3.77	0.00	25.00	0.10	50.08	0.03	-	-	-	-
	8	3.58	0.00	25.00	0.09	50.14	0.02	75.07	0.03	-	-
	16	3.28	0.00	24.98	0.11	50.02	0.09	62.16	0.02	75.00	0.03
50	0	7.36	0.07	52.00	0.81	-	-	-	-	-	-
	8	7.38	0.06	50.00	0.7	-	-	-	-	-	-
	16	7.31	0.06	49.99	0.90	-	-	-	-	-	-
70	0	10.27	0.15	70.03	1.55	-	-	-	-	-	-
	8	10.26	0.13	69.96	1.45	-	-	-	-	-	-
	16	10.30	0.10	69.92	1.75		-	-	-	-	-

**Table 4:** Summary table of vibration parameters of the test rig in the Z-axis.

f_0_ (Hz)	AoA (°)	f_1_ (Hz)	A_1_ (m/s^2^)	f_2_ (Hz)	A_2_ (m/s^2^)	f_3_ (Hz)	A_3_ (m/s^2^)	f_4_ (Hz)	A_4_ (m/s^2^)
25	0	3.54	0.00	25.00	0.10	50.00	0.02	-	-
	8	3.24	0.00	25.00	0.09	50.07	0.02	-	-
	16	3.71	0.00	24.98	0.12	50.02	0.01	75.00	0.01
50	0	7.36	0.02	50.02	0.70	-	-	-	-
	8	7.31	0.06	49.99	0.69	-	-	-	-
	16	7.31	0.02	49.99	0.67	57.03	0.12	-	-
70	0	10.27	0.45	70.03	0.65	-	-	-	-
	8	10.26	0.32	69.96	0.64	-	-	-	-
	16	10.30	0.23	69.92	0.82	-	-	-	-

## Data Availability

All data supporting the findings of this study are already included within the article.

## Abbreviations

The following abbreviations are used in this manuscript:

EPS	Electric propulsion systems
AoA	Angle of attack
SMA	Smart material alloys
FFT	Fast Fourier transform
PSD	Power spectral density
UAV	Unmanned Aerial Vehicle
NES	Nonlinear energy sink
SEM	Single equivalent mass
TMM	Transfer matrix method
SANC	Self-adaptive noise cancelation
CSCD	Cyclic symplectic component decomposition
CNT	Continuous wavelet transform
VS	Vibration sensor
AR	Auto regression
AE	Acoustic emission
CAD	Computer-aided design
FEM	Finite element method
CFD	Computational fluid dynamics
AC	Alternating current
PC	Personal computer
